# Towards a Discovery of a Zinc-Dependent Phosphate Transport Road in Plants

**DOI:** 10.3390/plants11223066

**Published:** 2022-11-12

**Authors:** Hui-Kyong Cho, Jaspreet Sandhu, Nadia Bouain, Chanakan Prom-u-thai, Hatem Rouached

**Affiliations:** 1 Plant Resilience Institute, Michigan State University, East Lansing, MI 48824, USA; 2 Department of Plant, Soil, and Microbial Sciences, Michigan State University, East Lansing, MI 48823, USA; 3 Department of Biochemistry and Molecular Biology, Michigan State University, East Lansing, MI 48823, USA; 4 Lanna Rice Research Center, Chiang Mai University, Chiang Mai 50200, Thailand; 5 Agronomy Division, Department of Plant and Soil Sciences, Faculty of Agricultural, Chiang Mai University, Chiang Mai 50200, Thailand

**Keywords:** phosphate, zinc, signaling crosstalk

## Abstract

Owing to the impending global scarcity of high-quality sources of phosphate (Pi) fertilizers, lowering its use in crop production requires improved insights into factors stimulating Pi uptake from the soil as well as the efficacious use by plants. Following decades of extensive research on plants’ adaptation to Pi deficiency with mitigated success in the field, a better understanding of how plants exposed to zinc (Zn) deficiency accumulate much more Pi provides a novel strategy in comparison to when plants are grown in Zn-rich soils. In this context, we review current knowledge and molecular events involved in the Pi and Zn signaling crosstalk in plants that will bear great significance for agronomical and rudimentary research applications.

## 1. Introduction

For their growth and development and to face changing environments, plants rely on a constant supply of phosphorus (P). Plant root systems primarily uptake P in their inorganic forms (Pi) [1,2,3,4,5], which are converted into vital molecules such as nucleic acids and energy. Ensuring efficient Pi levels in plants is a challenge for agronomy [1,2,3,4,5]. The use of P fertilizers is required to enhance the yield in fields [1,2,3,4,5]. Nevertheless, because P is a limited resource, the growing population is facing a potential food crisis. For this reason, a coordinated action to prevent a detrimental shortage of Pi is needed [6,7]. Researchers are constantly searching for new methods to enhance Pi rock extraction yield, reduce its chelation by metals in soil, and improve its uptake by plants.

In soil, Pi interacts with cations such as zinc (Zn) and forms immobilized complexes (i.e., Pi-Zn). Therefore, facilitating a progressive liberation of these elements into the soil and to avoid chelation/precipitation of Pi by Zn together with a constant improvement in fertilizer formulas are needed [8,9]. The existence of a strong interdependence between Pi and Zn in planta has been also reported by early agronomic/physiological studies [10]. Progress can now be made to decipher the molecular mechanisms used by plants to integrate Pi and Zn signals to control key physiological and developmental processes owing to the development of systems’ genetic approaches. In this review, we emphasize the homeostatic interactions between Pi and Zn to determine how Zn-deficiency signaling influences the accumulation of P in plants.

## 2. Pi Uptake Is the First Limiting Step for Pi Accumulation

In general, a significant amount of Pi is found in soils, although plants are only able to gain access to bioavailable Pi [5]. Plants have acquired highly regulated and efficient responses to optimize Pi acquisition in low Pi environments [5]. Under Pi deficiency, the plant activates the Pi starvation pathway and triggers a genome-wide gene expression reprograming which leads to the expression of various genes under the control of several transcription factors (TF), including the key TF PHOSPHATE STARVATION RESPONSE 1 (PHR1) [11,12,13]. This then leads to the fine tuning of the different processes including Pi metabolism and Pi transport in plants ranging from Pi uptake, loading into xylem, and root-to-shoot transfer to plant organs/cell organelles distribution [4].

When plants access high levels of Pi, for instance, after Pi replate, the cell metabolism quickly reverts to conditions that impede Pi uptake. We know that under these conditions (P-sufficiency) PHR1 interacts with proteins containing SPX domains [14], which involves inositol Pi compounds, reducing the pool of free PHR1. These observations clearly show that the plant tightly controls Pi homeostasis depending on P-deficiency and applies a high control over Pi uptake that depends on PHT1 regulation.

Stimulating Pi uptake without completely triggering the Pi starvation-signaling pathway could be used to improve plant growth capacity under a unfavorable nutritional (P) environment.

## 3. Zinc Starvation Bypasses the Pi-Deficiency Signaling Pathway

When identifying novel ways to regulate Pi uptake, it is important to avoid the classical comparison of Pi starvation versus Pi-rich conditions. Instead, future studies need to leverage intriguing observations where plants over-accumulate Pi during biotic or abiotic stresses, including in the presence of Pi concentrations that are elevated.

As aforementioned, Zn deficiency causes an overaccumulation of Pi. Notably, since the expression of Pi transporters is not induced by other macro- or micronutrients tested in several plant species, this Zn/Pi relationship appears to be specific [15]. The induction of the expression of genes encoding P uptake transporters under Zn deficiency has been reported in monocots and dicots [16,17,18,19,20]. Moderate Pi supply under low Zn can even lead to a limitation in biomass (due to excess internal Pi), which can only be reverted by adding Zn [20]. Conversely, applying Pi causes a decrease in Zn concentration in crop plants. This demonstrates that maintaining fertilization within a specific range is crucial for achieving satisfactory growth in low Zn conditions and that force-feeding plants with Pi could be counterproductive.

Investigations into the molecular basis of the Zn/Pi interaction in plants have begun recently, while the mechanisms explaining the unexpectedly enhanced *PHT1* expression in low Zn conditions remain poorly understood [21]. Recent work showed that the increased Pi content under low Zn requires PHR1 and PHO1, two core elements of the classical signaling pathway triggered by Pi starvation [10]. Perhaps a critical role in Pi transport under Zn deficiency is attributed to the Pi exporter PHO1;H3, which plays a negative regulatory role in Pi root-to-shoot under Zn deficient conditions, even though this process also requires PHO1 [10].

Importantly, it remains unknown why Pi uptake stimulation in roots remains activated in low Zn environments, even when internal Pi levels are high. The increase in Pi uptake due to Zn deficiency stimulates *PHT1* expression in the whole plant but mostly in the leaves, whereas Pi deficiency induces *PHT1* expression throughout the plant but predominantly in the roots. The Pi uptake stimulation triggered by Zn and Pi deficiencies appears to rely on the induction of *PHT1;1* for Zn deficiency [21,22], and primarily PHT1;1 and PHT1;4 for Pi starvation [3]. Furthermore, it remains to be shown whether the transcriptional activation of *PHT1;1* is solely responsible for the increased P uptake in low Zn, or if it also involves post-translational regulations.

## 4. The Growing Role of Lipids in Regulating Pi Homeostasis in Plants, but Which One?

**Figure 1:**
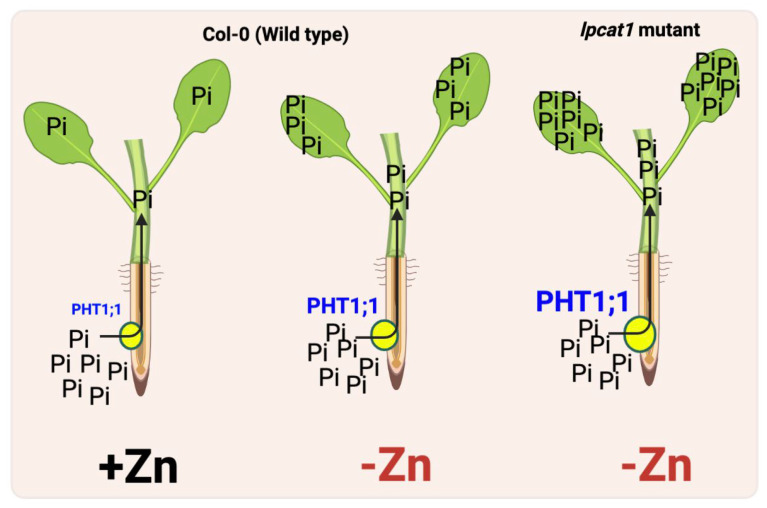
Schematic representation of signaling pathways controlling phosphate accumulation under zinc deficiency. In roots, phosphate (Pi) is acquired by high-affinity Pi transporters, PHT1;1. After following its radial transport, Pi is loaded into the xylem. Under −Zn conditions, the expression of *PHT1;1*, Pi uptake, and translocation to shoots increases in wild-type plants, which are further enhanced in the *Lysophosphatidylcholine* (*LPC*) *acyltransferase 1* (*LPCAT1*) mutant background. LPCAT1 regulates the LPC:phosphatidylcholine (PC) ratio, which, in turn, increases the expression of the Pi transporter *PHT1;1*, thereby resulting in the accumulation of Pi in shoots. Pi transfers to shoots, represented by a black arrow.

By employing a Genome-Wide Applications Studies (GWAS) analysis of the Pi content variation among *A. thaliana* accessions grown in a low Zn condition, Lyso-Phosphatidyl Choline AcylTransferase 1 (LPCAT1) proven to be a mitigating factor that curtails Pi accumulation and the induction of *PHT1;1* triggered by Zn deficiency [21] (Figure 1). However, the initial step leading to *PHT1;1* deregulation remains to be identified even though these studies highlight an important role for *PHT1;1* in the Zn deficiency response.

Our knowledge of phospholipids (PL)-derived signals in plants has been scant until the recent past; but physiological and molecular studies have demonstrated that some PL classes might act as precursors to generate diverse signaling molecules [22,23]. The fact is that *LPCAT1* mutation resulted in elevated *PHT1;1* expression level; and eventually, the over-accumulation of Pi under Zn deficiency provides compelling evidence supporting the role of a Lyso-PC/PC-derived signal in regulating Pi homeostasis under Zn deficiency. As a case in point, Lyso-PC has acted as a signal to regulate the expression of arbuscular mycorrhiza (AM)-specific Pi transporter genes in various plants species including *Lotus japonicus*, tomatoes, and potatoes [24,25]. Apart from the involvement of individual PLs in ion transport in plants, the emerging broader importance of changes in Lyso-PC/PC ratio to regulate plant development can be seen. For example, changing the Lyso-PC/PC ratio reduces the time to flower in Arabidopsis [26]. Another good example was observed in human cells where the Lyso-PC/PC ratio was linked to the inhibition of cell signaling, and metabolism [27,28,29]. Finally, exhibiting a link between lipid metabolism and Pi accumulation in Zn deficient condition, prepares the foundation to examine the role of lipid-derived signal in controlling ion homeostasis in plant cells as well as other organisms.

## 5. Conclusions

Nutrient–nutrient interaction along with its ensuing impact on their accumulation in plants has elicited widespread recognition. The interaction between Pi and Zn homeostasis demonstrates the way in which the deficiency of one element can lead to the over-accumulation of the other element. This observation opens up new avenues to take measures in making improvements in the plants’ Pi response. However, there is ambiguity on whether these responses have an underlying genetic/signaling or nutritional basis. However, except for the example of P/Zn interaction, more studies need to be conducted to understand how plants sense levels of nutrients not only in the surrounding soil but also within their cells before adjusting various transport steps that then lead to nutrient accumulation in cells. This is critically important to augment our existing knowledge of how plants regulate/coordinate these processes to facilitate the design of strategies to improve plant nutrition, particularly Pi nutrition.
